# Opportunistic computed tomography (CT) assessment of osteoporosis in patients undergoing transcatheter aortic valve replacement (TAVR)

**DOI:** 10.1007/s11657-025-01579-4

**Published:** 2025-07-17

**Authors:** Michael Paukovitsch, Tom Fechner, Dominik Felbel, Johannes Moerike, Wolfgang Rottbauer, Steffen Klömpken, Horst Brunner, Christopher Kloth, Meinrad Beer, Anjany Sekuboyina, Dominik Buckert, Jan S. Kirschke, Nico Sollmann

**Affiliations:** 1 Department of Cardiology, Ulm University Heart Center, Albert-Einstein-Allee 23, 89081 Ulm, Germany; 2 Department of Diagnostic and Interventional Radiology, University Hospital Ulm, Albert-Einstein-Allee 23, 89081 Ulm, Germany; 3 MoMan - Center for Translational Imaging, University Hospital Ulm, Einstein-Allee 23, 89081 Ulm, Germany; 4 Department of Informatics, Information and Technology, TUM School of Computation, Technical University of Munich, Arcisstr. 21, 80333 Munich, Germany; 5 Department of Diagnostic and Interventional Neuroradiology, School of Medicine and Health, TUM Klinikum Rechts der Isar, Technical University of Munich, Ismaninger Str. 22, 81675 Munich, Germany; 6 TUM-Neuroimaging Center, TUM Klinikum Rechts der Isar, Technical University of Munich, 81675 Munich, Germany; 7 Department of Nuclear Medicine, University Hospital Ulm, Albert-Einstein-Allee 23, 89081 Ulm, Germany

**Keywords:** Osteoporosis, Computed tomography, Transcatheter aortic valve replacement, Opportunistic screening, Artificial intelligence

## Abstract

**Summary:**

CT-based opportunistic screening using artificial intelligence finds a high prevalence (43%) of osteoporosis in CT scans obtained for planning of transcatheter aortic valve replacement. Thus, opportunistic screening may be a cost-effective way to assess osteoporosis in high-risk populations.

**Background:**

Osteoporosis is an underdiagnosed condition associated with fractures and frailty, but may be detected in routine computed tomography (CT) scans.

**Methods:**

Volumetric bone mineral density (vBMD) was measured in clinical routine thoraco-abdominal CT scans of 207 patients for planning of transcatheter aortic valve replacement (TAVR) using an artificial intelligence (AI)-based algorithm.

**Results:**

43% of patients had osteoporosis (vBMD < 80 mg/cm^3^ L1-L3) and were elderly (83.0 {interquartile range [IQR]: 78.0–85.5} vs. 79.0 {IQR: 71.8–84.0} years, *p* < 0.001), more often female (55.1 vs. 28.8%, *p* < 0.001), and had a higher Society of Thoracic Surgeon’s score for mortality (3.0 {IQR:1.8–4.6} vs. 2.1 {IQR: 1.4–3.2}%, *p* < 0.001). In addition to lumbar vBMD (58.2 ± 14.7 vs. 106 ± 21.4 mg/cm^3^, *p* < 0.001), thoracic vBMD (79.5 ± 17.9 vs. 127.4 ± 26.0 mg/cm^3^, *p* < 0.001) was also significantly reduced in these patients and showed high diagnostic accuracy for osteoporosis assessment (area under curve: 0.96, *p* < 0.001). Osteoporotic patients were significantly more often at risk for falls (40.4 vs. 22.9%, *p* = 0.007) and required help in activities of daily life (ADL) more frequently (48.3 vs. 33.1%, *p* = 0.026), while direct-to-home discharges were fewer (88.8 vs. 96.6%, *p* = 0.026). In-hospital bleeding complications (3.4 vs. 5.1%), stroke (1.1 vs. 2.5%), and death (1.1 vs. 0.8%) were equally low, while in-hospital device success was equally high (94.4 vs. 94.9%, *p* > 0.05 for all comparisons). However, one-year probability of survival was significantly lower (84.0 vs. 98.2%, log-rank *p* < 0.01).

**Conclusion:**

Applying an AI-based algorithm to TAVR planning CT scans can reveal a high rate of 43% patients having osteoporosis. Osteoporosis may represent a marker related to frailty and worsened outcome in TAVR patients.

**Graphical Abstract:**

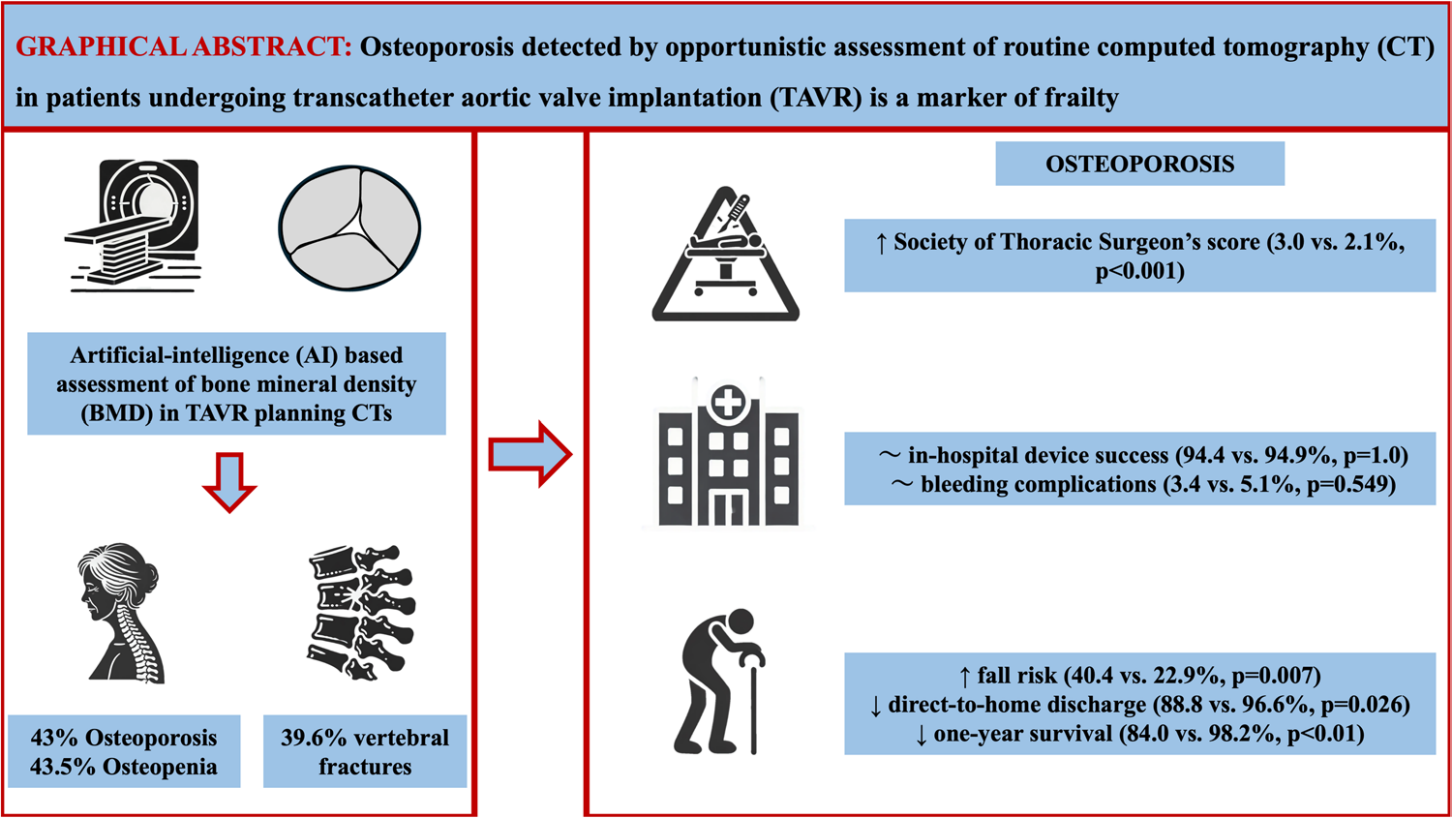

**Supplementary Information:**

The online version contains supplementary material available at 10.1007/s11657-025-01579-4.

## Introduction

Transcatheter aortic valve replacement (TAVR) has proven its safety and efficacy in treating patients with aortic stenosis (AS) with low, intermediate, and high surgical risk [1–4]. Typically, AS is a condition seen in elderly patients with various comorbidities [5].

Similar to AS, the prevalence of impaired bone quality relating to reduced bone mineral density (BMD) and osteoporosis increases with age and has an estimated prevalence of 21.7% among elderly patients worldwide [6]. Many of those elderly patients suffer from a decline in physical, psychological, and social functions often summarized as frailty [7], which is associated with increased mortality after cardiac procedures [8] and TAVR [9]. Frailty is a very common finding among patients with osteoporosis [10] and AS [11] and these populations may likely overlap. However, osteoporosis is considered an underdiagnosed condition worldwide and a severe health problem due to frequent hip or especially vertebral fractures with increased morbidity and mortality [12, 13]. This is mainly due to the observation that modalities such as dual-energy absorptiometry (DXA) or dedicated quantitative computed tomography (QCT) are often only performed when fragility fractures have already occurred, thus leading to a high socio-economic burden [14, 15].

With the advancements in image processing and artificial intelligence (AI), CT scans obtained during clinical routine may, however, be used for determining BMD, thus allowing opportunistic screening for osteoporosis in clinical routine CT scans specifically obtained for indications other than assessing BMD [16–19]. An AI‐based architecture has been developed that allows opportunistic extraction of volumetric BMD (vBMD) after fully automated vertebral body labeling and segmentation in routine non-contrast or contrast‐enhanced CT [16, 17, 19]. It has shown high diagnostic yield as it improved the discrimination of osteoporotic vertebral fractures as compared to routine DXA as well as dedicated QCT [16, 18]. Patients scheduled for TAVR undergo thoraco-abdominal CT scans for procedural planning and prostheses selection, which can be exploited for opportunistic vBMD assessments without additional radiation exposure or costs.

Against this background, we aimed to determine vBMD, prevalence, and clinical impact of osteoporosis in TAVR patients using an AI-based algorithm on clinical routine CT scans for TAVR planning.

## Methods

### Study population and TAVR procedures

All patients provided written informed consent for data collection as part of the Ulm University Heart Center TAVR registry (approved by the local institutional review board). This study complies with the principles of the Declaration of Helsinki. This was a retrospective, monocenter study including 207 consecutive patients who underwent TAVR for treatment of AS at the Ulm University Heart Center between March 2021 and June 2022. Patients were eligible for TAVR based on the heart team’s consensus after evaluation of age and comorbidities as outlined in current guidelines [20].

All TAVR procedures were performed using fluoroscopy guidance and under mild conscious sedation as well as local anesthesia of puncture sites. Femoral access was used for introduction of the TAVR delivery sheath. Ahead of TAVR procedures, patients received a standardized thoraco-abdominal CT scan (one to five days ahead of TAVR), which included the thoraco-lumbar spine and was used for opportunistic vBMD assessment for the present study. Dedicated software (3Mensio Structural Heart, version 10.2; Pie Medical, Maastricht, The Netherlands) was used for detailed planning of TAVR procedures.

For 207 out of 245 patients treated with TAVR during the inclusion time frame, CT datasets were available for assessment of vBMD. In 29 out of 245 patients, CT scans were performed at external hospitals and could not be processed further for vBMD analysis due to unavailability or technical reasons (such as insufficient resolution with a slice thickness > 1 mm as a requirement for data input in the AI-based algorithm). For 9 patients, vBMD of the lumbar region (the reference standard used in this study according to American College of Radiologists [ACR] criteria [21]) could not be determined due to vertebral fractures. Besides patients for whom sufficient CT data for vBMD analysis was unavailable, no further exclusion criteria existed.

For analysis of the patients’ functional status, physician and nursing examination records were accessed, which included fall risk assessment or assessment of help required in activities of daily life (ADL). A patient was classified showing fall risk if one of the following criteria applied: a history of falling, use of ambulatory aid (such as crutches, cane, walker, nurse assist, furniture), or impaired (difficulty rising from chair/head down when walking with poor balance/requires walking aid or needs to hold on to furniture) or weak (stooped but able to lift head while walking, patient uses short steps when walking) gait/transferring. Discharge records were accessed to determine the discharge destination of a patient (e.g., to an external hospital or nursing home). One-year patient follow-up examination was conducted by out-patient visits or telephone follow-up calls (if a patient did not appear for the respective out-patient visit). If one-year follow-up data were not available, the longest available follow-up interval was used for those patients.

### Definitions

Osteoporosis was defined according to recommendations by the ACR as an average vBMD ≤ 80 mg/cm^3^ in the lumbar vertebrae (L1 to L3) and osteopenia as an average vBMD ≤ 120 mg/cm^3^ and > 80 mg/cm^3^ [21]. Technical success and in-hospital device success after TAVR were determined according to Valve Academic Research Council-3 (VARC-3) criteria [22].

### Imaging by CT

Clinical routine CT scans for TAVR planning were performed in the supine position with a dual-source CT or single-source CT system (Somatom Force, N = 191, or Somatom Definition AS +, N = 16; Siemens Healthineers, Erlangen, Germany). Each examination was divided into two scans, first an electrocardiography (ECG)-triggered scan of the heart, immediately followed by an untriggered scan of the aorta including the pelvic axis, both in arterial contrast phase. Intravenous contrast agent was applied during scanning (Ultravist 300 [Bayer Vital GmbH, Leverkusen, Germany] was used as the standard contrast agent; in few cases, Imeron 400 [Bracco Imaging GmbH, Konstanz, Germany] was also used, depending on the pump setup). The amount of contrast medium was administered in a body mass index (BMI)-adapted manner with increasing flow rates (BMI < 25 kg/m^2^: 56 ml, 3 flow; BMI 25–30 kg/m^2^: 68 ml, 4 flow; BMI > 30 kg/m^2^: 80 ml, 5 flow).

The CT scan was started by bolus triggering in the thoracic aorta with a Hounsfield unit (HU) threshold of 100 and a delay of 5 s. The scans were performed in helical mode with a tube voltage between 70 and 130 kV (in 10 kV intervals) and the use of tube current modulation (70 kV, N = 39; 80 kV, N = 74; 90 kV, N = 44; 100 kV, N = 41; 110 kV, N = 1, 120 kV, N = 7, 130 kV, N = 1). The slice thickness was 0.75 mm or 1 mm, reconstructed with a soft tissue kernel.

### CT-based assessment of vBMD

**Fig. 1:**
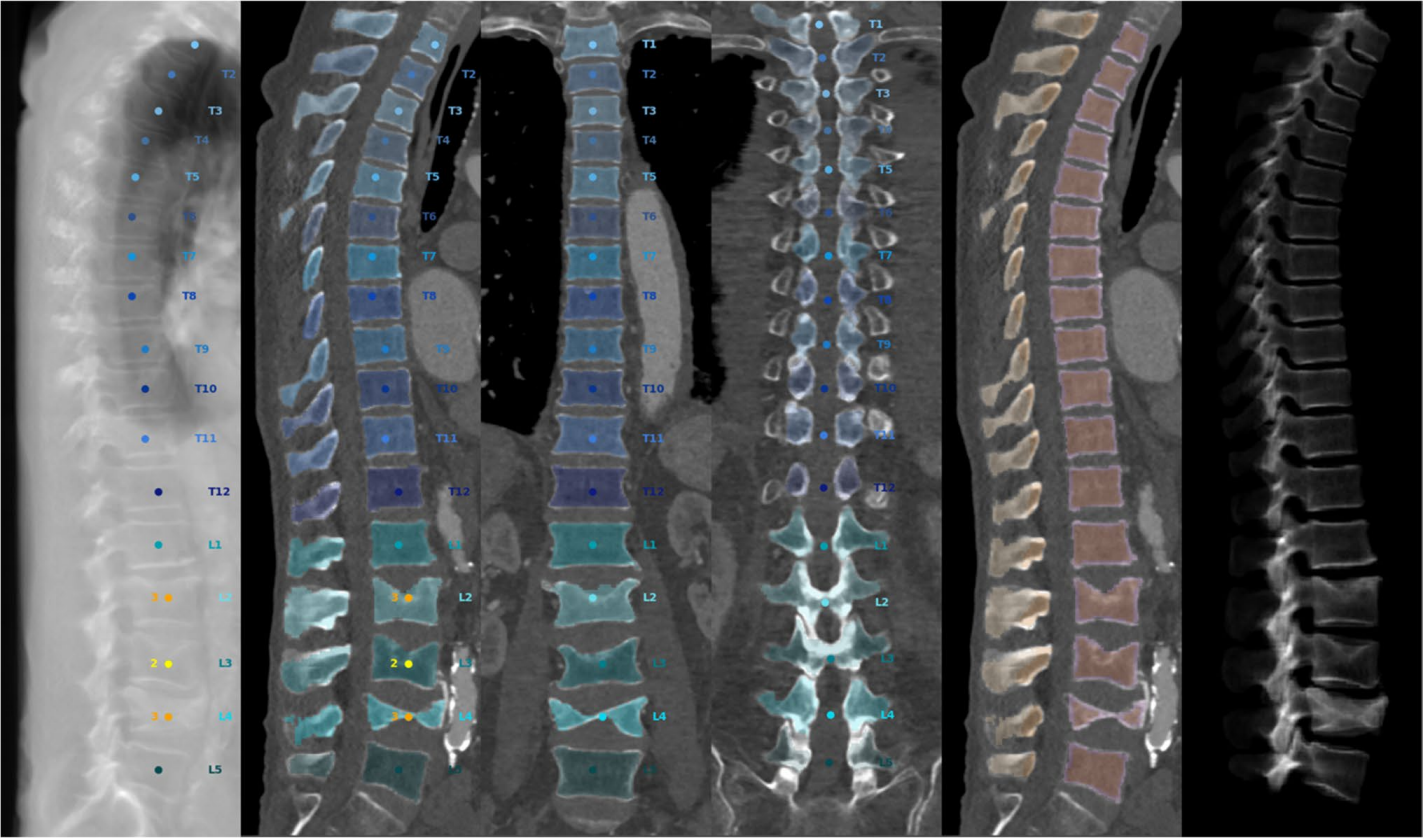
Automated spine labeling and segmentation of the thoraco-lumbar spine (T1-L5) in a routine TAVR planning CT using coronal and sagittal views, together with virtual radiographs in sagittal view. This patient had three lumbar vertebral fractures (L2-L4, Genant grades 2 and 3)

Contrast-enhanced clinical routine CT scans for TAVR planning were used to opportunistically determine vertebral vBMD using SpineQ software (version 1.0; Bonescreen GmbH, Munich, Germany). The CT scans for opportunistic measurements and associated Digital Imaging and Communications in Medicine (DICOM) metadata were exported from the Picture Archiving and Communication System (PACS) and uploaded and processed in the software’s environment. Using this AI-based algorithm with a convolutional neural network (CNN)-based architecture, automated spine detection, vertebral body labeling, vertebral body segmentations, and generation of trabecular subregion masks were facilitated, enabling the assessment of vBMD from clinical routine CT scans [15–19]. Moreover, in the context of vBMD extraction, the software corrects for the presence of contrast media and the contrast media phase using linear equations, thus enabling processing of contrast-enhanced clinical routine CT scans [15–17, 19]. The AI-based algorithm has previously been validated against reference standard DXA as well as QCT and was found to have high agreement with QCT- or DXA-derived measurements [16]. The vBMD measurements from this algorithm performed better in predicting fractures compared to QCT and DXA [16, 19]. All labelings and segmentations were manually checked for quality, and any insufficiently segmented vertebrae were excluded from vBMD extraction (Fig. 1).

Asynchronous calibration was used to convert HU into vBMD. The method of asynchronous calibration used a preexisting fixed relation between HU and vBMD, specific for a distinct type of CT scanner and scan protocol. This supersedes the need for placing a calibration phantom underneath a patient, which is required in conventional QCT scans used for assessment of osteoporosis. The relations and ensuing equations for conversion of HU to vBMD applied in asynchronous calibration have been previously obtained from scans of a density reference phantom for the scanner used in this study. Specifically, calibration factors were obtained by asynchronous phantom measurements with a QSA-717 phantom (QRM Quality Assurance in Radiology and Medicine GmbH, Möhrendorf, Germany) with different hydroxyapatite inserts.

### CT-based assessment of vertebral fractures and degenerative changes

The presence and level of vertebral fractures was determined visually by image review by a radiologist and fractures were further graded according to Genant et al. [23]. Individual vertebrae with fractures Genant grade ≥ 1 were excluded from vBMD assessments [17]. Furthermore, degenerative changes such as osteophytes can cause inaccuracy of vBMD measurements [17, 24]. Hence, such changes should therefore not be included. Degenerative changes were classified as previously described using a three-scale grading system (0 = none, I–III = mild, mild-to-severe, and severe) [17]. Any vertebrae with degenerations ≥ grade II were excluded from vBMD analysis. Overall, 3188 out of 3519 theoretically available (T1-L5) vertebrae were included for vBMD analysis.

### Statistical analysis

The patient population was analyzed in total as well as grouped according to the presence of osteoporosis (vBMD ≤ 80 mg/cm^3^ vs. vBMD > 80 mg/cm^3^ in L1-L3) and vertebral fractures (Genant grades ≥ 1 vs. no vertebral fracture; Supplementary Table 1). Categorical variables are shown as counts and percentages. Numerical variables are shown as mean ± standard deviation or median and interquartile range (IQR), if they were found to be non-normally distributed. Distribution of variables was analyzed using histograms and Q-Q plots.

Categorical variables were compared using the Chi-squared or the Fisher’s exact test, if > 20% of cells had expected frequencies below five. Numerical variables were compared using the *t*-test and the Mann–Whitney U test for non-normally distributed variables.

To identify predictors of osteoporosis, univariate and multivariate binary logistic regression analyses were used. Univariate logistic regression analysis was performed for all variables that significantly differed between groups. Variables significant in univariate logistic regression were further analyzed using multivariate binary logistic regression with a backwards likelihood inclusion preset. Multicollinearity was tested before inclusion in the multivariate model, and a Pearson and Spearman correlation coefficient of *r* ≥ 0.6 and a variance inflation factor ≥ 10 were used as a prespecified threshold. The EuroScoreII and Society of Thoracic Surgeons (STS) score were not included in the multivariate model for obvious autocorrelation as both scores include variables such as age and sex. Separate multivariate logistic regression models (adjusted for covariates from univariate logistic regression) were calculated for thoracic vBMD as well as thoraco-lumbar vBMD. Results of logistic regression were shown as odds ratio (OR) and its 95% confidence interval (CI).

Furthermore, the diagnostic accuracy of thoraco-lumbar and thoracic vBMD for detection of osteoporosis was analyzed using a receiver operating characteristics (ROC) analysis. Osteoporosis as defined by the ACR guidelines (vBMD in L1-L3 ≤ 80 mg/cm^3^) was used as the reference standard for this analysis [21]. Results of the ROC analysis are shown as area under curve (AUC) and its respective 95% CI. Optimal diagnostic cut-off values for thoraco-lumbar and lumbar vBMD were calculated using the Youden’s index. Furthermore, AUC was calculated for the vBMD reference interval previously published by Rühling et al. using the same AI-based algorithm for vBMD extractions [17]. For correlation analysis of vBMD across different vertebral regions, Pearson’s correlation index and its respective 95% CIs were calculated. One-year survival was compared using Kaplan–Meier curves and differences in survival were tested using the log-rank test.

All statistical testing was performed two-sided using SPSS software (version 29; IBM Corp., Armonk, NY, USA), and a *p*-value < 0.05 was considered statistically significant.

## Results

### Patient demographics, TAVR outcomes and frailty

Two hundred-seven patients were included in the final analysis. According to ACR criteria of reduced vBMD (< 80 mg/cm^3^) in L1-L3, 89/207 (43%) of patients in this study had osteoporosis (Table 1), 90/207 (43.5%) had osteopenia, and only 28/207 (13.5%) had normal vBMD. Results of the univariate and multivariate analyses are shown in Tables 2 and 3.

**Table 1:** Characteristics of patients without and with ACR-defined osteoporosis (mean lumbar vBMD ≤ 80 g/cm^3^)

Parameter	Total (*N* = 207)	No Osteoporosis (*N* = 118)	Osteoporosis (*N *= 89)	*p*
Mean vBMD (T1-L5), mg/cm^3^	101.1 ± 31.5	121.6 ± 23.6	74.0 ± 31.5	** < 0.001**
Mean vBMD (L1-L3), mg/cm^3^	85.7 ± 30.4	106.3 ± 21.4	58.2 ± 14.7	** < 0.001**
Mean vBMD (T1-T12), mg/cm^3^	106.8 ± 33.0	127.4 ± 26.0	79.5 ± 17.9	** < 0.001**
Vertebral Fracture(s)	82 (39.6)	31 (26.3)	51 (57.3)	** < 0.001**
Age, years	81.0 {75.0–84.0}	79.0 {71.8–84.0}	83.0 {78.0–85.5}	**0.001**
BMI, kg/m^2^	27.9 ± 5.0	28.5 ± 5.1	27.0 ± 4.8	**0.043**
Female, N (%)	83 (40.1)	34 (28.8)	49 (55.1)	** < 0.001**
aHT, N (%)	182 (87.9)	106 (89.8)	76 (85.4)	0.332
CAD, N (%)	134 (64.7)	79 (66.9)	55 (61.8)	0.443
Diabetes mellitus, N (%)	60 (29.9)	43 (36.4)	17 (19.1)	** < 0.001**
AFib, N (%)	80 (38.6)	37 (31.4)	43 (48.3)	**0.013**
NYHA II, N (%)	26 (12.6)	14 (11.9)	12 (13.5)	0.621
NYHA III, N (%)	151 (72.9)	87 (73.7)	64 (71.9)	
NYHA IV, N (%)	28 (13.5)	15 (12.7)	13 (14.6)	
Euro SCORE II, %	2.8 {1.8–5.1}	2.4 {1.6–4.2}	3.8 {2.1–6.8}	** < 0.001**
STS Score of Mortality, %	2.5 {1.5–3.2}	2.1 {1.4–3.2}	3.0 {1.8–4.6}	** < 0.001**
Troponin T, ng/L	26.0 {16.0–42.0}	28.0 {15.0–43.0}	25.0 {16.3–38.5}	0.330
NT-proBNP, pg/mL	1095.0 {451.5–3814.5}	1085.5 {455.3–2762.5}	1149.0 {424.0–4618.0}	0.332
Hb, g/dl	13.0 ± 1.8	13.2 ± 1.8	12.8 ± 1.7	0.141
eGFR, ml/min	62.1 ± 20.1	63.3 ± 19.6	60.4 ± 20.7	0.316
LV EF, %	55.1 ± 10.4	55.0 ± 10.0	55.2 ± 10.8	0.886
AV mPG, mmHg pre	44.1 ± 17.3	44.5 ± 17.2	43.6 ± 17.5	0.714
AV maxPG, mmHg pre	70.0 ± 26.8	69.7 ± 26.3	70.4 ± 27.6	0.868
sPAP, mmHg (N = 129)	44.2 ± 17.1	43.0 ± 17.6	45.6 ± 16.4	0.392
Fall Risk, N (%)	63 (30.4)	27 (22.9)	36 (40.4)	**0.007**
Requiring help in ADL, N (%)	82 (39.6)	39 (33.1)	43 (48.3)	**0.026**
Direct to home discharge, N (%)	193 (93.2)	114 (96.6)	79 (88.8)	**0.026**
**Type of implanted valve**				
BEV (Sapien platform)	108 (52.2)	64 (54.2)	44 (49.4)	0.494
SEV (Core Valve platform)	99 (47.8)	54 (45.8)	45 (50.6)	
Technical success	207 (100)			
In-hospital device success	196 (94.7)	112 (94.9)	84 (94.4)	1.0
**In-hospital complications**				
Bleeding (Major, Minor)	9 (4.4)	6 (5.1)	3 (3.4)	0.549
In-hospital death	2 (1.0)	1 (0.8)	1 (1.1)	1.0
Stroke	4 (1.9)	3 (2.5)	1 (1.1)	0.636
Pacemaker implantation	19 (9.2)	14 (11.9)	5 (5.6)	0.149

Values are shown as mean ± standard deviation or median {interquartile range} for continuous variables and absolute numbers (%) for dichotomous variables. Bold *p*-values indicate significant results

*vBMD* volumetric bone mineral density, *BMI* body mass index, *aHT* arterial hypertension, *CAD* coronary artery disease, *AFib* atrial fribrillation, *NYHA* New York Heart Association class, *STS* Society of Thoracic Surgeons, *NT-proBNP* N-terminal pro brain natriuretic peptide, *Hb* hemoglobin, *eGFR* estimated glomerular filtration rate, *AV* aortic valve, *mPG* mean pressure gradient, *maxPG* maximum pressure gradient, *sPAP* systolic pulmonary artery pressure, *BEV* balloon-expandable valve, *SEV* self-expandable valve, *ADL* activities of daily life, ACR *American College of Radiologists*

**Table 2:** Clinical predictors of ACR-defined osteoporosis (vBMD L1-L3 ≤ 80 g/cm^3^)

	Univariate		Multivariate	
	OR {95% CI}	***p***	OR {95% CI}	***p***
Mean vBMD (T1-L5), mg/cm^3^	0.81 {0.76–0.87)	** < 0.001**		
Mean vBMD (T1-T12), mg/cm^3^	0.87 {0.83–0.91}	** < 0.001**		
Age, years	1.1 {1.02–1.1}	**0.004**	1.04 {0.99–1.1}	0.074
BMI, kg/m^2^	0.94 {0.89–0.99}	**0.045**	0.97 {0.9–1.0}	0.258
Diabetes mellitus	0.41 {0.22–0.78}	**0.007**	0.49 {0.25–0.96}	**0.038**
Female	3.0 {1.7–5.4}	** < 0.001**	2.6 {1.4–4.6}	**0.002**
AFib	2.0 {1.2–3.6}	**0.014**	1.7 {0.92–3.1}	0.091
EuroScore II	1.1 {1.0–1.2}	**0.025**		
STS Score of Mortality	1.3 {1.1–1.5}	**0.002**		

Values are shown as odds ratio (OR) and respective 95% confidence intervals (CIs). Bold *p*-values indicate significant results. Variables differing in descriptive statistics between osteoporotic and non-osteoporotic patients were tested in univariate and multivariate logistic regression analyses using osteoporosis as the dependent variable to determine the relevant clinical covariates associated with osteoporosis. The EuroScoreII and STS Score for Mortality were not tested in multivariate regression due to autocorrelation with other variables such as age and sex, which are included in those scores

*AFib* atrial fibrillation, *vBMD* volumetric bone mineral density, *STS* Society of Thoracic Surgeons, *ACR* American College of Radiologists

**Table 3:** Multivariate analysis of predictors of ACR-defined osteoporosis (vBMD L1-L3 ≤ 80 g/cm^3^)

	Thoraco-lumbar vBMD (T1-L5) adjusted for clinical predictors			Thoracic vBMD (T1-T12) adjusted for clinical predictors		
	OR	95% CI	***p***	OR	95% CI	***p***
Mean vBMD (T1-L5), mg/cm^3^	0.77	0.71–0.84	**< 0.001**			
Mean vBMD (T1-T12), mg/cm^3^				0.85	0.81–0.9	**< 0.001**
Age, years	0.91	0.83–1.004	0.059	0.94	0.87–1.01	0.093
Female	4.2	1.07–16.2	**0.039**	2.5	0.83–7.3	0.104

Values are shown as odds ratio (OR) and respective 95% confidence intervals (CIs). The left half of the table shows thoraco-lumbar vBMD, the right half thoracic vBMD, adjusted for clinical covariates using multivariate logistic regression. The clinical covariates were derived from the multivariate analysis. Bold *p*-values indicate significant results

*ACR* American College of Radiologists, *vBMD* volumetric bone mineral density

Patients with osteoporosis were significantly elderly (83.0 {IQR: 78.0–85.5} vs. 79.0 {IQR: 71.8–84.0} years, *p* < 0.001) and more often female (55.1 vs. 28.8%, *p* < 0.001). Interventional risk was significantly higher both according to the STS score for mortality (3.0 {IQR:1.8–4.6} vs. 2.1 {IQR: 1.4–3.2} %, *p* < 0.001) as well as the EuroScoreII (3.8 {IQR: 2.1–6.8} vs. 2.4 {IQR: 1.6–4.2} %, *p* < 0.001). The BMI was significantly lower in patients with osteoporosis (27.0 ± 4.8 vs. 28.5 ± 5.1 kg/m^2^, *p* = 0.043), whereas the prevalence of diabetes mellitus was also lower (19.1 vs. 36.4%, *p* < 0.001). Symptom burden measured as New York Heart Association (NYHA) class was similar between patient groups (*p* = 0.621; Table 1). In-hospital technical success was achieved in all patients. In-hospital device success was equally high among patients with and without osteoporosis (94.9 vs. 94.4%, *p* = 1.0). Moreover, the rate of major complications such as in-hospital death (1.1 vs. 0.8%, *p* = 1.0), stroke (1.1 vs. 2.5%, *p* = 0.636), and bleeding (3.4 vs. 5.1%, *p* = 0.549) did not significantly differ between patient groups.

Despite similar in-hospital outcome, patients with osteoporosis were significantly more often deemed to be at risk for falls (40.4 vs. 22.9%, *p* = 0.007) and required help in activities of daily life (ADL) significantly more frequently (48.3 vs. 33.1%, *p* = 0.026). These patients required discharge to an external hospital (e.g., geriatric hospital) or nursing facility more often, thus the rate of direct discharge home was significantly lower (88.8 vs. 96.6%, *p* = 0.026).

### Lower probability of 1-year survival in patients with osteoporosis

**Fig. 2:**
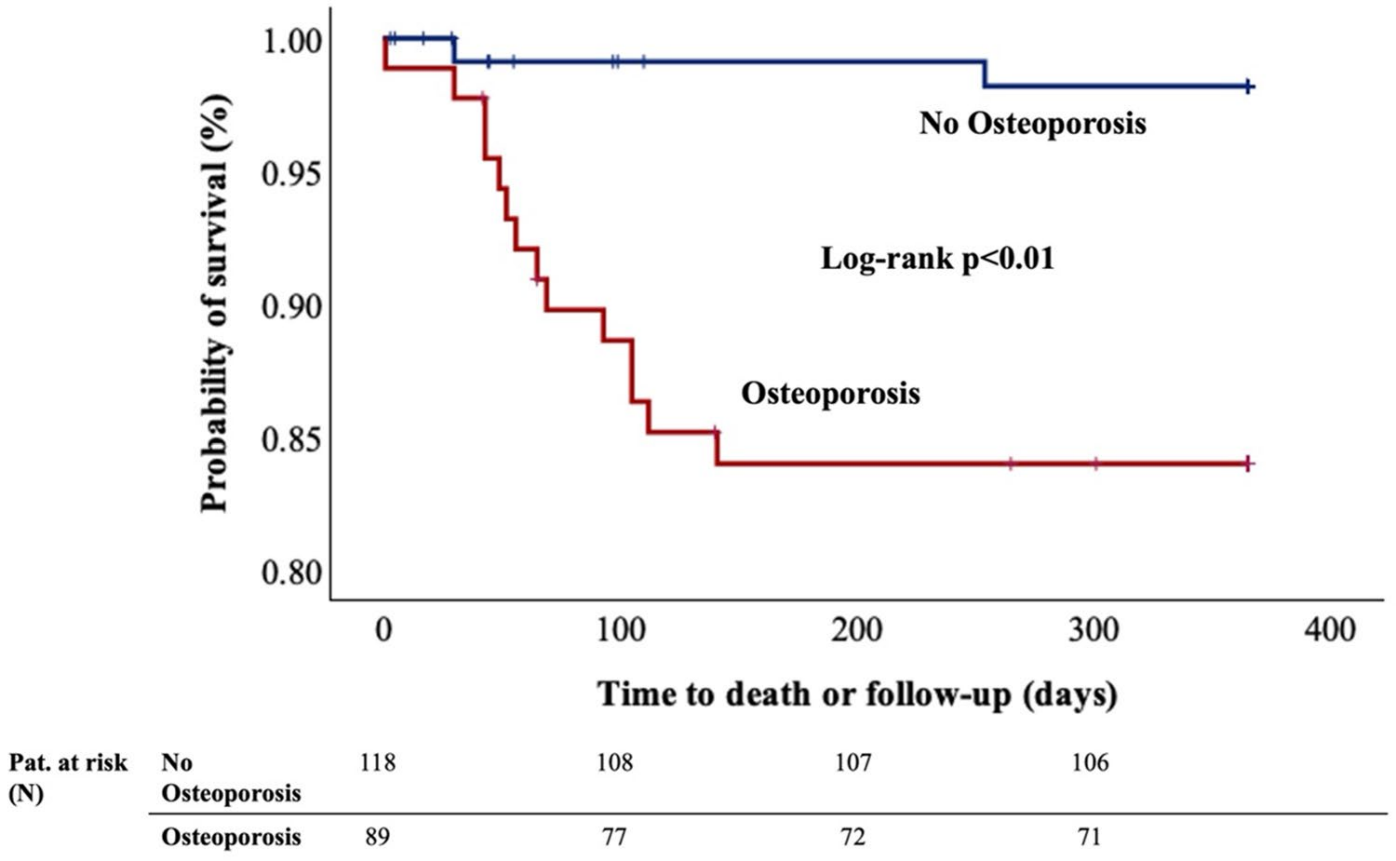
Kaplan–Meier analysis for probability of survival comparing patients with (red) and without osteoporosis (blue). The log-rank test revealed a significant difference in one-year survival (*p* < 0.01)

92.8% of all patients completed the one-year follow-up interval with a similar distribution among those with and without osteoporosis (94.4 vs. 91.5%, *p* = 0.59). Kaplan–Meier analysis revealed a significant difference in one-year survival (log-rank *p* < 0.01) (Fig. 2). Probability of one-year survival was 84.0% in osteoporotic patients compared to 98.2% in those patients without osteoporosis.

### vBMD across different regions of the spine

In addition to lumbar vBMD in L1-L3 (58.2 ± 14.7 vs. 106 ± 21.4 mg/cm^3^, *p* < 0.001), thoracic vBMD in T1-T12 (79.5 ± 17.9 vs. 127.4 ± 26.0 mg/cm^3^, *p* < 0.001) was also significantly reduced in patients with osteoporosis. Moreover, vBMD was highly correlated among vertebral regions T1-T12 and L1-L3 (*r* = 0.89 {IQR: 0.86–0.92}, *p* < 0.001) in the overall patient population. Lumbar vBMD in L1-L5 (96.2 ± 11.2 vs. 138.5 ± 12.6 mg/cm^3^, *p* < 0.001) as well as thoracic vBMD in T1-T12 (118.3 ± 19.1 vs. 156.7 ± 23.5 mg/cm^3^, *p* < 0.001) were significantly reduced likewise when comparing patients with osteopenia to those patients without osteoporosis or osteopenia (Supplementary Table 1).

Mean thoracic vBMD (T1-T12) showed high diagnostic accuracy (Table 4) for assessing osteoporosis according to ACR criteria (AUC: 0.96 {95% CI: 0.93–0.98}, *p* < 0.001). According to the Youden’s index, the cut-off with highest sensitivity and specificity was 103.9 mg/cm^3^ with a sensitivity of 95.5% and specificity of 82.2% in this population. A cut-off of 100 mg/cm^3^ yielded high diagnostic accuracy as well (AUC: 0.86 {95% CI: 0.8–0.91}, *p* < 0.001) with sensitivity and specificity of 86.5% and 84.7%, respectively.

**Table 4:** Diagnostic accuracy for predicting ACR-defined osteoporosis with vBMD of different vertebral regions

	AUC {95% CI}	***p***	Optimal cut-off*	Sensitivity	Specificity
Mean thoraco-lumbar vBMD (T1-L5), mg/cm^3^	0.98 {0.96–0.99}	**< 0.001**	93.5	90%	93.2%
Mean thoracic vBMD (T1-T12), mg/cm^3^	0.96 {0.93–0.98}	**< 0.001**	103.9	95.5%	82.2%
Mean Thoracic vBMD (T1-T12) < 100 mg/cm^3 †^	0.86 {0.8–0.91}	**< 0.001**		86.5%	84.7%

Area under the curve (AUC) and respective 95% confidence intervals {95% CIs} are shown. Bold *p*-values indicate significant results. * according to maximum Youden’s index; †according to Rühling et al. (https://doi.org/10.1007/s00330-022-08721-7)

*ACR* American College of Radiologists, *vBMD* volumetric bone mineral density

Both thoracic vBMD (OR: 0.95 {95% CI 0.81–0.9}, *p* < 0.001) and thoraco-lumbar vBMD (OR: 0.77 {0.71–0.84}, *p* < 0.001) were found to significantly associate with osteoporosis in multivariate logistic regression adjusted for clinical covariates. For this analysis, clinical covariates (Table 2) were identified from univariate and multivariate regression and entered into separate multivariate regression models with thoracic and thoraco-lumbar vBMD (Table 3).

### Vertebral fractures

The overall prevalence of vertebral fractures amounted to 39.6%, and fractures were significantly more frequently detected in patients with osteoporosis (57.3 vs. 26.3%, *p* < 0.001). Overall, 21.3% had one, whereas 7.7% had two and 10.6% of the studied population had ≥ 3 fractured vertebrae. The subgroup analysis of patients with fractures (Supplementary Table 2) confirmed lower thoraco-lumbar vBMD in patients with fractures (86.8 ± 30.0 vs. 110 ± 30.7 mg/cm^3^, *p* < 0.001) as well as lower lumbar vBMD in L1-L3 (71.3 ± 27.4 vs. 95.0 ± 28.6 mg/cm^3^, *p* < 0.001) and lower thoracic vBMD in T1-T12 (92.5 ± 28.4 vs. 116.2 ± 32.5 mg/cm^3^, *p* < 0.001). Diagnostic accuracy for assessment of fractures was fair for lumbar vBMD (L1-L3, AUC: 0.73 {95% CI: 0.66–0.8}, *p* < 0.001) as well as for thoracic vBMD (T1-T12, 0.71 {95% CI: 0.64–0.78}, *p* < 0.001; Supplementary Table 3).

Patients with vertebral fractures were also significantly elderly compared to those without fractures (83.0 {IQR: 76.3–86.0} vs. 79.9 {IQR: 75.0–84.0}, *p* < 0.007). The rates of patients classified as having fall risk (40.2 vs. 24.0%, *p* = 0.013) and requiring help in ADL (48.8 vs. 33.6%, *p* = 0.029) were likewise found to be significantly higher in patients with fractures.

## Discussion

In this study, 207 consecutive patients treated with TAVR for AS underwent opportunistic assessment of vBMD using an AI-based algorithm for analysis of clinical routine CT scans for TAVR planning with the following main findings:

- 43% of patients had osteoporosis, whereas 43.5% of patients had osteopenia according to opportunistic vBMD assessment.
- Osteoporosis may be related to frailty in TAVR patients as those were found to require help in ADL and were at risk of falls more often. Consecutively, direct-to-home discharge was less frequently observed in osteoporotic patients. Moreover, probability of one-year survival was significantly worse in patients with osteoporosis (98.2 vs. 84.0%, *p* < 0.01).
- In-hospital outcomes of TAVR measured as technical and device success as well as bleeding and stroke rates were similar between patients with and without osteoporosis, despite greater interventional risk according to the STS score for mortality in patients with osteoporosis.
- Both thoracic and the overall thoraco-lumbar vBMD showed high diagnostic accuracy for opportunistically assessing osteoporosis.

Previous studies investigating osteoporosis [25–29] or BMD [30] in TAVR patients relied mostly on patient records [26–29] or surrogate formulas [25, 26] to identify patients at risk for osteoporosis [26, 27], but did not investigate the prevalence of osteoporosis itself. Saji et al. used an osteoporosis self-assessment tool (OST) based on a formula incorporating the patients’ age and weight to identify patients at risk for osteoporosis [26]. Whereas 92.4% of patients were found to be at high risk for osteoporosis in their study, osteoporosis had only been diagnosed in 23.2% before TAVR and even fewer received medication for osteoporosis [26]. In another study by Saji et al., a preexisting diagnosis of osteoporosis was noted in health records of 27.6% of patients [27], with a significantly higher prevalence of 42.5% among patients with fractures [27]. The multicenter Women’s International Transcatheter Aortic Valve Implantation (WIN-TAVI) registry was specifically set up to investigate outcomes and comorbidities in women undergoing TAVR [29]. This registry enrolled 1019 women with only 17.5% having a history of osteoporosis in patient records [29]. Those studies have in common that they used self-assessment approaches [25–27] or patients’ records [29] to identify patients with osteoporosis. However, osteoporosis is an underdiagnosed condition worldwide, and there is a diagnostic gap regarding detecting osteoporosis before fractures occur and a patient may become symptomatic [12, 13]. Hence, deriving information about osteoporosis from patients’ records harbors the risk of considerably underestimating the fraction of patients having osteoporosis. With the herein used approach of opportunistic vBMD assessment using an AI-based algorithm with clinical routine CT scans for TAVR planning, accurate determination of osteoporosis can become feasible without additional examinations, thus keeping clinical management efforts, image-based radiation dose exposures, and extra costs low whilst extracting valuable add-on information from preexisting imaging data. In contrast, Demirel et al. analyzed bone in patients undergoing TAVR by measuring HU of thoracic vertebrae and the sternum on TAVR CT scans [30]. Low HU measurements were associated with increased mortality in their study [30]. Solla-Surez et al. similarly used HU and found that osteosarcopenia, but not reduced vertebral bone density itself, was associated with increased mortality [31]. However, HU measurements may not be used as a proxy for actual vBMD values, and thus may not be used for defining osteoporosis or osteopenia since bone CT attenuation can vary considerably depending on factors like peak tube voltage or intravenous contrast media, amongst other factors [32, 33]. Bone CT attenuation was measured significantly higher in contrast-enhanced phases when compared to a non-contrast phase, and measurements in the arterial and portal phases resulted in up to 25% false negatives [33]. Consequently, when determining accurate vBMD values, correction for determinants such as the distinct scanning protocol and presence of contrast media is necessary, and this could be achieved by the AI-based algorithm used in this study.

Compared to 43% of patients having osteoporosis in our current study, the low rates of osteoporosis previously reported in TAVR populations [26, 29] seem to confirm that osteoporosis is an underdiagnosed condition and potentially insufficient approaches have been used for accurately detecting osteoporosis. This observation has been well established for the general population [12], but is now also explicitly described in TAVR patients. Given that AS requiring a TAVR procedure is often a condition of elderly patients, a high prevalence of osteoporosis may be expected if specific screening exams were conducted. In this regard, DXA measurement of areal BMD (aBMD) is still the most common exam for screening and diagnosing osteoporosis [34]. However, in a global survey, the International Society for Clinical Densitometry (ISCD) and the International Osteoporosis Foundation (IOF) concluded that there is significant variability in the access to and quality of DXA services [14]. Additionally, screening rates seem to be low even in high-risk patient groups such as elderly patients with fractures and glucocorticoid users [35].

Opportunistic screening for vBMD in CT scans using an established AI-based algorithm may help in closing the existing diagnostic gap, which is of high clinical relevance. Specifically, it is known that osteoporosis is underdiagnosed worldwide [12, 13]. The major reason behind this gap is that BMD assessments by DXA or QCT are commonly only performed when fragility fractures have already occurred. However, opportunistic assessments using non-dedicated CT data from clinical routine that have been acquired for other purposes than osteoporosis screening (e.g., staging in oncologic patients or for TAVR planning like in the present study) have the potential of identifying the undiagnosed patients early, therefore helping to initiate fracture prevention therapies that otherwise would not be realized in the same proportion of patients [15, 36–38]. Hence, opportunistic screening for vBMD may have direct clinical impact when performed during future clinical routine to reduce morbidity and mortality, given that respective vBMD information could be used as an add-on report to raise awareness of bone health for clinicians. In this regard, vBMD derived from CT may be more appropriate for assessing osteoporosis when compared to DXA-based aBMD, given that it has been suggested that DXA can be inaccurate in differentiating between patients with and without prevalent vertebral fractures, in predicting new fragility fractures, and for therapy monitoring [39–41]. Furthermore, opportunistic screening using CT with vBMD extraction is a cost-effective approach and may reduce the need for additional radiologic exams with x-ray exposure [42]. Yet, it is important that opportunistically derived vBMD values are accurate and may be derived from any vertebrae, given that clinical routine scans may considerably vary in spatial coverage of the spine. In this context, our study showed high correlation between thoracic und lumbar vBMD, confirming the findings of a previous study [17]. Consecutively, thoracic vBMD yielded high diagnostic accuracy for osteoporosis, which is commonly diagnosed using only L1-L3 according to ACR recommendations [21]. However, vBMD derived from thoracic vertebrae could considerably extent the range of CT scans usable for opportunistic screening (e.g., in a cardiac CT or CT coronary angiography).

Fractures are the main consequences of osteoporosis and are associated with negative health impact [43]. A relevant number of fractures occur soon (within 12 months) after the initial diagnosis of osteoporosis [44], which emphasizes the need for early-on treatment and monitoring of reduced vBMD. In our study, 39.6% of patients had vertebral fractures, hence these patients were identified at an already progressed stage of the disease. Yet, with opportunistic assessments of osteoporosis using clinical routine CT scans such as those for TAVR planning, detection of reduced bone density may become possible also in early stages, thus enabling identification of patients at risk for future fragility fractures. Vertebral fractures represent the most frequently occurring fractures in osteoporosis, and affected patients have a significantly increased risk of future additional fractures [45]. This relates to high morbidity, mortality, and socio-economic burden [12, 13]. Hence, early intervention is required in risk populations to prevent first or additional fractures, which could be facilitated also among patients treated with TAVR for AS.

In the context of comorbidities observed in TAVR patients, osteoporosis could be regarded another condition linked to frailty, such as low BMI [46] or sarcopenia [47]. This seems in line with previous studies, which have demonstrated a link between osteoporosis and frailty [48, 49]. However, it needs to be emphasized that osteoporosis may overlap with frailty, but it is not the same as frailty itself, which may be constituted of a combination of several factors and not osteoporosis alone. Despite the greater frailty burden and higher interventional risk according to the STS score for mortality, the rates of in-hospital device success, bleeding, and stroke did not significantly differ between osteoporotic and non-osteoporotic patients in our cohort. Contrarily, probability of survival was significantly lower in osteoporotic patients in our study.

Due to a lack of studies comparing in-hospital adverse events in osteoporotic patients undergoing TAVR, a direct comparison across cohorts is not possible. Considering the impact of frailty, in-hospital complications such as bleeding, in-hospital mortality, and stroke have been reported to be similar in sarcopenic and non-sarcopenic patients [47], and frailty did not predict the 30-day composite endpoint (including death, stroke, myocardial infarction, and life-threatening bleeding) in the WIN-TAVI registry [29]. Frailty has, however, been associated with in-hospital major bleeding complications after TAVR in the Frailty Aortic Valve Replacement (FRAILTY-AVR) study [50]. Moreover, frailty [9, 11, 51] and its surrogates such as low BMI [46] and sarcopenia [47] have been associated with increased long-term mortality [9, 11, 46, 51] and rehospitalization after TAVR [51]. Considering this evidence, TAVR may show an acceptable in-hospital safety profile in patients with osteoporosis, similar to what is known from studies investigating patient characteristics associated with frailty [9, 11, 51].

### Limitations

Our findings are limited to early outcomes after TAVR. Moreover, site-specific treatment standards may influence in-hospital outcome and complications, thus making findings not fully generalizable. A more comprehensive geriatric assessment and use of clinical frailty scales such as the Essential Frailty Toolset (EFT) are not standard clinical practice in patients who undergo TAVR. Data from such assessments could possibly yield further insights into the role of reduced vBMD and osteoporosis in the larger context of frailty and associated comorbidities. In this context, frailty was not directly assessed in this study, but osteoporosis may be considered a surrogate condition based on its established associations with frailty [48, 49]. Probability of survival was lower in patients with osteoporosis; however, the higher comorbidity burden in these patients, reflected for instance by the higher STS score, may act as a confounder. Due to the limited sample size presented in our study, multivariate analysis to adjust for confounders was not performed.

## Conclusion

Opportunistic assessment of vBMD using TAVR planning CT scans from clinical routine with an AI-based algorithm yielded a high rate of 43% of patients having osteoporosis. Vertebral fractures were observed in 57.3% of patients with osteoporosis, emphasizing an already progressed disease in these patients. Further, vBMD values derived from thoracic and lumbar vertebrae were highly correlated and thoracic vBMD accurately determined osteoporosis. This suggests the feasibility of vBMD assessments in CT scans containing only the thoracic vertebrae, which would allow low-cost osteoporosis screening in even larger cardiologic patient populations. Automated vBMD assessment may thus become the standard-of-care for many of the CT scans performed in everyday clinical practice. Osteoporosis may be linked to frailty in TAVR patients who have lower rates of direct-to-home discharges, require help in ADL more often, and have reduced probability of one-year survival.

## Supplementary Information

Below is the link to the electronic supplementary material.

Supplementary Material 1(DOCX 20.7 KB)

## Data Availability

All relevant data is included within the manuscript and its supplements.
