# Supplementary material for: Opportunistic computed tomography (CT) assessment of osteoporosis in patients undergoing transcatheter aortic valve replacement (TAVR)
Source: Arch Osteoporos. 2025 Jul 17;20(1):100. doi: 10.1007/s11657-025-01579-4 (PMC12271236; doi:10.1007/s11657-025-01579-4)
Supplement: Supplementary file 1 — Supplementary Material 1(DOCX 20.7 KB) [file 11657_2025_1579_MOESM1_ESM.docx]

# Supplemental Material

| **Supplemental Table 1. vBMD grouped according to the presence of osteopenia excluding patients with osteoporosis** | | | | |
| --- | --- | --- | --- | --- |
|  | **Total**  **(N=118)** | **No Osteopenia**  **(N=28)** | **Osteopenia**  **(N=90)** | **p-Value** |
| vBMD (L1-L3), mg/cm^3^ | 106.2±21.4 | 138.5±12.6 | 96.2±11.2 | **<0.001** |
| vBMD (T1-T12), mg/cm^3^ | 127.4±26.0 | 156.7±23.5 | 118.3±19.1 | **<0.001** |
| vBMD (T1-L5), mg/cm^3^ | 1221.6±25.6 | 151.3±19.0 | 112.4±16.0 | **<0.001** |
| Values are shown as mean±standard deviation. Bold p-values indicate significant results.  Abbreviations: vBMD = volumetric bone mineral density | | | | |

| **Supplemental Table 2. Characteristics of patients with and without vertebral fractures** | | | |
| --- | --- | --- | --- |
| **Parameter** | **No Fracture**  **N=125** | **Fracture**  **N=82** | **p-Value** |
| Mean vBMD (T1-L5), mg/cm^3^ | 110.6±30.7 | 8.8±30.0 | **<0.001** |
| Mean vBMD (L1-L3), mg/cm^3^ | 95.0±28.6 | 71.3±27.4 | **<0.001** |
| Mean vBMD (T1-T12), mg/cm^3^ | 116.2±32.5 | 92.5±28.4 | **<0.001** |
| Normal BMD | 24 (19.2) | 4 (4.9) | **<0.001** |
| Osteopenia | 63 (50.4) | 27 (32.9) |  |
| Osteoporosis | 38 (30.4) | 51 (62.2) |  |
| Age, years | 79.0 {75.0-84.0} | 83.0 {76.3-86.0} | **0.007** |
| BMI, kg/m^2^ | 27.8±5.1 | 27.9±4.8 | 0.869 |
| Female, N (%) | 49 (39.2) | 34 (41.5) | 0.745 |
| aHT, N (%) | 111 (88.8) | 71 (86.6) | 0.632 |
| CAD, N (%) | 79 (63.2) | 55 (67.1) | 0.568 |
| Diabetes mellitus, N (%) | 42 (33.6) | 18 (22.0) | 0.071 |
| AFib, N (%) | 43 (34.4) | 37 (45.1) | 0.121 |
| NYHA II, N(%) | 20 (16.0) | 6 (7.3) | 0.137 |
| NYHA III, N (%) | 85 (68.0) | 66 (80.5) |  |
| NYHA IV, N (%) | 18 (14.4) | 10 (12.2) |  |
| Euro SCORE II, % | 2.7 {1.8-4.8} | 3.0 {2.0-5.4} | 0.430 |
| STS Score Risk of Mortality, % | 2.3 {1.6-3.6} | 2.8 {1.6-4.1} | 0.487 |
| Troponin T, ng/L | 28.0 {16.0-43.5} | 24.0 (15.5-39.0} | 0.261 |
| NT-proBNP, pg/mL | 1105.0 {483.0-3845.0} | 1095.0{396.0-3658.0} | 0.948 |
| Hb, g/dl | 12.9±1.9 | 13.0±1.6 | 0.624 |
| eGFR, ml/min | 61.1±21.2 | 63.5±18.4 | 0.417 |
| LV EF, % | 55.1±10.3 | 55.2±10.6 | 0.909 |
| AV mPG, mmHg pre | 45.9±18.9 | 41.5±14.3 | 0.080 |
| AV maxPG, mmHg pre | 73.6±28.3 | 64.7±23.8 | **0.024** |
| sPAP, mmHg  N=129 | 44.0±15.9 | 44.3±18.8 | 0.937 |
| Fall Risk, N (%) | 30 (24.0) | 33 (40.2) | **0.013** |
| Requiring help in ADL, N (%) | 42 (33.6) | 40 (48.8) | **0.029** |
| Direct to home discharge, N (%) | 6 (4.8) | 8 (9.8) | 0.165 |
| **Type of implanted valve** |  |  |  |
| BEV (Sapien platform) | 69 (55.2) | 39 (47.6) | 0.282 |
| SEV (Core Valve platform) | 56 (44.8) | 43 (52.4) |  |
| Technical success | 125 (100) | 82 (100) | 1.0 |
| In-hospital device Success | 119 (95.2) | 76 (92.7) | 0.547 |
| **In-hospital complications** |  |  |  |
| Bleeding (Major, Minor) | 7 (5.6) | 2 (2.4) | 0.488 |
| In-hospital death | 0 | 2 (2.4) | 0.156 |
| Stroke | 3 (2.4) | 1 (1.2) | 1.0 |
| Pacemaker implantation | 14 (11.2) | 5 (6.1) | 0.214 |
| Values are shown as mean±standard deviation or median {interquartile range) for continuous variables and absolute number (%) for dichotomous variables. Bold p-values indicate significant results.  Abbreviations: vBMD = volumetric bone mineral density; BMI = body mass index; aHT = arterial hypertension; CAD = coronary artery disease; MI = myocardial infarction; AFib = atrial fribrillation; NYHA = New York Heart Association class; STS = Society of Thoracic Surgeons; NT-proBNP = N-terminal pro brain natriuretic peptide; Hb = hemoglobin, eGFR = estimated glomerular filtration rate; AV = aortic valve; mPG = mean pressure gradient; maxPG = maximum pressure gradient; sPAP = systolic pulmonary artery pressure; BEV = balloon-expandable valve; SEV = self-expandable valve | | | |

| **Supplemental Table 3. Diagnostic accuracy of vBMD at different vertebral levels for fractures** | | | | | |
| --- | --- | --- | --- | --- | --- |
|  | **AUC {95% CI}** | **p** | **Optimal cut-off*** | **Sensitivity** | **Specificity** |
| Mean vBMD (T1-L5), mg/cm^3^ | 0.72 {0.65-0.79} | **<0.001** | 109.1g/cm3 | 84.1% | 48.8% |
| Mean vBMD (L1-L3), mg/cm^3^ | 0.73 {0.66-0.8} | **<0.001** | 87.5g/cm3 | 77.8% | 57.6% |
| Mean vBMD (T1-T12), mg/cm^3^ | 0.71 {0.64-0.78} | **<0.001** | 113.9g/cm3 | 80.1% | 50.4% |
| Area under curve (AUC) and respective 95% confidence intervals {95% CI} are shown. Bold p-values indicate significant results. * according to maximum Youden’s index  Abbreviations: vBMD = volumetric bone mineral density | | | | | |
